# Cardioprotective mIGF-1/SIRT1 signaling induces hypertension, leukocytosis and fear response in mice

**DOI:** 10.18632/aging.100464

**Published:** 2012-06-11

**Authors:** Giulia Bolasco, Raffaele Calogero, Matteo Carrara, Mumna Al Banchaabouchi, Daniel Bilbao, Gianluigi Mazzoccoli, Manlio Vinciguerra

**Affiliations:** ^1^ European Molecular Biology Laboratory (EMBL)-Mouse Biology Unit, Monterotondo, Italy; ^2^ Molecular Biotechnology Center, University of Turin, Italy; ^3^ Department of Medical Sciences, Division of Internal Medicine and Chronobiology Unit, IRCCS Scientific Institute “Casa Sollievo della Sofferenza”, San Giovanni Rotondo, Italy; ^4^ Institute of Hepatology, Birkbeck University of London, UK

**Keywords:** behaviour, blood pressure, IGF-1, immune system, SIRT1

## Abstract

Locally acting insulin growth factor isoform (mIGF-1) and the NAD+-dependent protein deacetylase SIRT1 are implicated in life and health span. Heart failure is associated with aging and is a major cause of death. mIGF-1 protects the heart from oxidative stresses via SIRT1. SIRT1 subcellular localization and its genomic regulation by mIGF-1 are unknown. We show here that SIRT1 is located in the nuclei of a significant fraction of cardiomyocytes. Using high throughput sequencing approaches in mIGF-1 transgenic mice, we identified new targets of the mIGF-1/SIRT1 signaling. In addition to its potent cardioprotective properties, cardiac-restricted mIGF-1 transgene induced systemic changes such as high blood pressure, leukocytosis and an enhanced fear response, in a SIRT1-dependent manner. Cardiac mIGF-1/SIRT1 signaling may thus modulate disparate systemic functions.

## INTRODUCTION

Cardiovascular diseases increase during aging and are first cause of mortality, representing one third of all global deaths and a major burden for the health systems. Insulin like growth factor-1 (IGF-1) and Sirtuin-1 (SIRT1) are key mediators of cell homeostasis and of cardiac stress; moreover they have been implicated in biological aging processes [1-6]. Mammals display a complex IGF-I signaling system with multiple alternative spliced isoforms that have distinct effects on cardiovascular function [1, 7, 8]. These splicing variants share a common core peptide, flanked by varying termini (Class 1 and 2 N-terminal peptides, and E peptides). IGF-I can operate both as a systemic growth factor produced by the liver in response to growth hormone and as a local growth factor acting in an autocrine/paracrine manner in organs such as the heart. The locally acting mIGF-I isoform comprises Class 1 N-terminal and Ea C-terminal peptides [1, 7, 8]. Mouse genetics studies have shown that enhancement of the mIGF-I signaling pathway is highly effective in promoting skeletal muscle regeneration, cell survival and renewal [7]. mIGF-I transgenic overexpression throughout postnatal life does not result in perturbation of cardiac physiology and is able to recover heart function after harsh injuries that trigger heart failure [9], which is mediated by the inflammatory response. mIGF-I enhances antioxidative cell defenses by up-regulating genes that display anti-oxidant and anti-apoptotic properties [9]. Also, mIGF-I repairs the heart from injury through production of specific cytokines that cross-talk with the bone marrow and recruit endothelial-primed cells for de novo vascularization of the myocardial tissue, indicating that cardiomyocyte specific overexpression of this transgene can have profound systemic effects [10].

The Sirtuin family of nicotinamide adenine dinucleotide (NAD+)-dependent protein deacetylases plays a role in the regulation of organism healthspan [2]. SIRT1 is the largest and best characterized Sirtuin and its activation displays pleiotropic beneficial effects [2]. Pharmacological or nutritional interventions capable of activating SIRT1 enzymatic activity have been shown to increase life span in model organisms [11-13]. In contrast, SIRT1 knock-out (KO) mice die at birth or soon after [14, 15]. Moderate SIRT1 overexpression protected mice from cardiac oxidative stress and postponed the onset of age-dependent cardiac fibrosis and cell death [16]. In vitro and in vivo findings corroborated these cardio-protective effects of SIRT1, suggesting that SIRT1 activation might be of benefit for the treatment of cardiac diseases [2][17][18]. SIRT1 transgenic over-expression or knock-out in various cell types has surpri-singly been shown to modulate the production of specific cytokines and systemic inflammatory responses [19].

IGF-I and SIRT1 can impinge on the same signaling pathways [1]. In this respect, we have shown that the IGF-I core protein isoform and the locally acting mIGF-I isoform display distinct effects in the protection from cardiac stress [5]. Using hypertrophic/oxidative stressors, we identified a signaling pathway that protects cardiomyocytes and which relies on the activation of SIRT1 by the locally acting mIGF-I isoform [5]. Heart-specific transgenic mIGF-I mice displayed a 2-fold increase in SIRT1 expression levels compared to their wild type littermates, and this was paralleled with a down-regulation in the acetylation levels of SIRT1 substrates (H1, p53) in the hearts [5]. Accordingly, in vitro mIGF-I overexpression protected cardiomyocytes from cell injuries induced by hypertrophic and oxidative stress in a specific SIRT1-dependent manner, through activation of anti-oxidant genes [5]. Noteworthy, the circulating IGF-I core peptide did not modulate SIRT1 expression and/or activity and it was not beneficial during stress conditions [5]. Recently, we generated cardiac-specific mIGF-I transgenic mice in which SIRT1 was excised from adult cardiomyocytes in an inducible (tamoxifen) and conditional fashion (mIGF-1 Tg × SirT1 CKO) to better assess the role of mIGF-I/SIRT1 signaling in vivo. Analysis of these animals confirmed that mIGF-I-induced SIRT1 activity is required to protect the heart from oxidative stress-induced cell damage and lethality [6].

The robust responses obtained with mIGF-I-induced SIRT1 activity suggested a potential mechanistic basis for strategies to improve the outcome of heart disease, and we were interested in further defining the molecular mechanisms involved in this process.

It is known that SIRT1 can deacetylate both cytoplasmic and nuclear proteins and it binds chromatin to regulate gene expression [20]; however there is no information about the localization and the transcription/chromatin dynamics of mIGF-1-activated SIRT1 in the heart.

While addressing these issues using localization and high throughput sequencing approaches, we uncovered a surprising systemic role for the cardiac mIGF-1/SIRT1 pathway in mice, which has a global impact on the regulation of the immune system, the arterial blood pressure and also the behavioral response to fear.

## RESULTS

### SIRT1 is localized in the nucleus of cardiomyocytes, but not of non cardiac cell types

**Figure 1:**
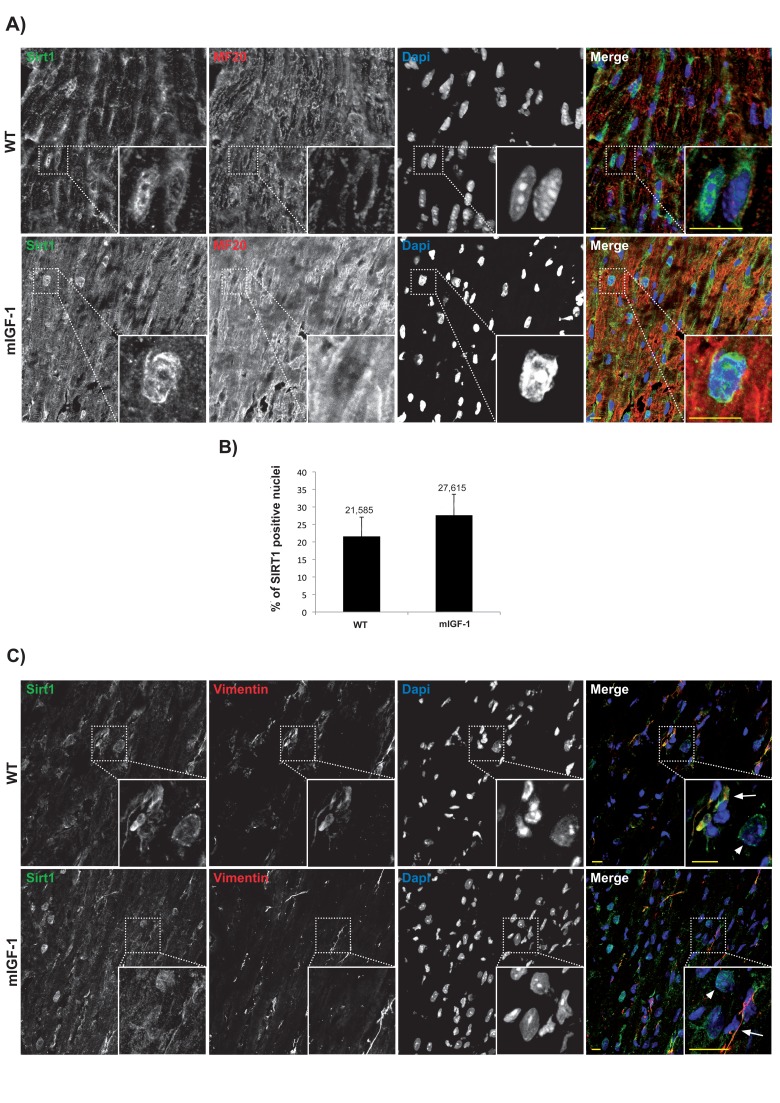
Confocal analysis of SIRT1 localization in the heart of wild type and mIGF-1 transgenic mice (**A**) representative heart sections of a 3 months old wild type (WT, upper panel) and of a cardiac restricted mIGF-1 transgenic mouse (mIGF-1, lower panel), stained for cardiomyocyte specific marker sarcomeric myosin (red), SIRT1 (green). Nuclei are counterstained with DAPI (blue). (**B**) quantification of SIRT1 positive nuclei, as shown in **A**. Total DAPI positive nuclei considered for quantification of SIRT1 immunopositivity in WT = 2280, and in mIGF-1 Tg = 2053, in 4 independent preparations. (**C**) representative heart sections of a 3 months old WT (upper panel) and of a cardiac restricted mIGF-1 Tg mouse (lower panel), stained for vimentin (red), SIRT1 (green). Nuclei are counterstained with DAPI (blue). Mesenchymal cells cytoplasm (arrows) appeared positive for both vimentin (red) and SIRT1 (green). Whereas in other cell types, presumably cardiomyocytes due to nuclear localization of SIRT1 (green), vimentin is lacking in thecytoplasm (arrowheads). Scale Bars= 10 μm.

Subcellular localization of chromatin remodeling SIRT1 varies in different cell types, and it is crucial for its impact on cellular functions. Previous reports based mainly on biochemical fractionation techniques indicated that SIRT1 is localized in the cytoplasm of cardiomyocytes in basal conditions, and nucleo-cytoplasmic shuttling is a regulatory mechanism of SIRT1 that may participate in the stress response [21, 22]. While the cardiomyocytes constitute the bulk of heart mass, this organ is composed only for ~60% of this cell type, and fibroblasts and other cell types (endothelial cells, macrophages, stem cells) account for the remaining fraction [23]. A bidirectional cross-talk between cardiomyocytes and non-cardiac cells is crucial to respond to pathological stimuli [23], and SIRT1-dependent metabolic effects may be cell- and compartment specific. To date, a quantitative microscopy-based approach to elucidate cell-specific SIRT1 localization in the heart has not been undertaken. Aiming to this, we first evaluated SIRT1 localization in the cardiomyocytes. Confocal analyses of heart cryo-sections from unstimulated 3 months old wild type (WT) and mIGF-1 transgenic (Tg) mice were performed. Sections were co-stained with SIRT1 (Fig.1 Panel A and C, green), and a specific marker of cardiomyocyte sarcomeric myosin (MF-20, Fig.1 Panel A and C, red), and DAPI counterstaining was used for the nuclei (Fig.1 Panel A and C, blue). Software-assisted analysis showed that SIRT1 was localized in ~22 % of the cardiomyocytes nuclei in WT mice and in ~28 % of the cardiomyocytes nuclei in mIGF-1 Tg mice (Fig. 1A and B). Co-staining for SIRT1 and vimentin (class III intermediate filament, marker of fibroblasts and mesenchymal like cells) revealed an exclusive cytoplasmic co-localization of SIRT1 in these non cardiac cells both in WT and mIGF-1 mice (Fig. 1, Panel C). Typically, larger SIRT1 positive nuclei in vimentin negative cells (Fig. 1 Panel C, arrowheads), presumably belonging to cardiomyocytes, were found adjacent to vimentin positive cells (Fig. 1 Panel C, arrows). SIRT1 was found exclusively in the cytoplasm also in endothelial cells, as determined by co-localization with CD144 (vascular endothelial-cadherin) (data not shown). These findings suggest that, in the heart of both WT and mIGF-1 mice, SIRT1 localizes in the nucleus of a substantial fraction of cardiomyocytes, whereas it is only cytoplasmic in non cardiac cell types.

### High throughput sequencing analyses of cardiac mIGF-1/SIRT1 signaling

**Figure 2:**
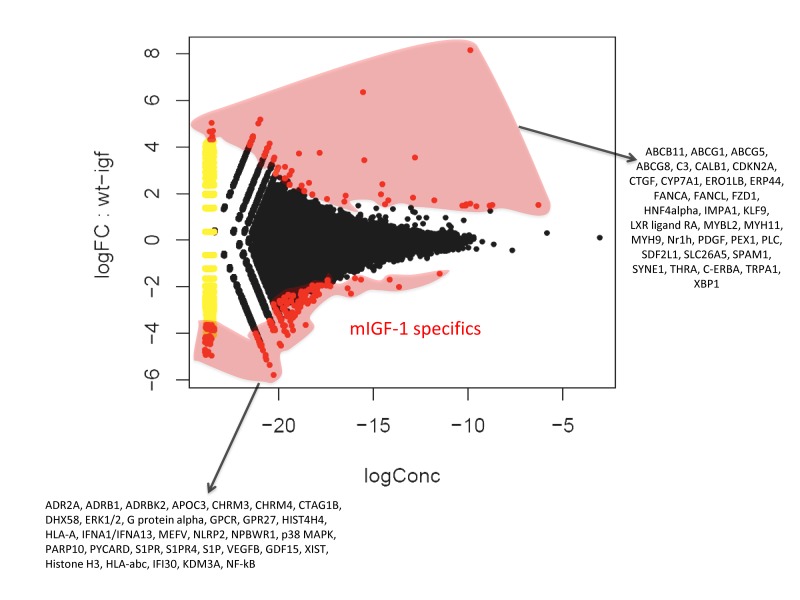
Fold change (FC) plot using common dispersion The hearts of 2 WT and 2 cardiac-restricted mIGF-1 Tg mice were excised, chromatin was extracted and immunoprecipitated with an anti-SIRT1 antibody. Purified DNA fragments were processed for sequencing with a Solexa Gene Analyzer II platform (Illumina). Upon bioinformatic analyses, 302 TSS were identified as SIRT1 DNA-bound. Red dots represent TSS differentially bound by SIRT1 in the WT or in the mIGF-1 Tg background, while black dots represent TSS bound similarly by SIRT1 in either genotype.

Chromatin immunoprecipitation followed by sequencing (ChIP-Seq) methodology [24] has been used to show that SIRT1 binds to highly repetitive genomic DNA and to a functionally diverse subset of genes in embryonic stem cells [20]. To our knowledge this is the only data set available on SIRT1 genome-wide binding. In view of the fact that we found SIRT1 localized in 22 to 28% of cardiomyocytes nuclei, with the aim of understanding the nuclear genomic function of this deacetylase, we performed genome-wide profiling of its binding to DNA in the hearts of WT or mIGF-1 Tg mice by using ChIP-Seq and a specific ChIP-grade commercial antibody. The experiments were run in duplicate for both genotypes and between 78 to 95% of the reads were mapped. The majority of SIRT1 counts localized to repetitive DNA regions (data not shown) in agreement with previous studies [20]. We selected those localized within 10 Kb from gene start site (TSS) (from www.ensembl.org). 302 SIRT1 bound TSS were identified, corresponding to 183 functional genes, 68 pseudogenes and 61 predicted genes. As shown in Fig. 2 by a common dispersion plot, while the majority of the DNA SIRT-1 bound genes were the same between WT and mIGF-1 Tg mice, a subset of genes were differentially bound by SIRT1 in the heart of either WT or mIGF-1 Tg mice. Clustering these genes according to biological functions, SIRT1 differentially bound TSS of established key players of cardiovascular function/blood pressure (ABCG1, C3, CTGF, MYH11, PDGF, TRPA1 in WT hearts; and ADRB1, ERK1/2, G protein alpha, GPCR, p38 MAPK, VEGFB, NF-kB in mIGF-1 Tg hearts), of atherosclerosis/inflammation/oxidative stress (CDKN2A, ABCG5 ABCG8, CYP7A1, ERO1LB, ERP44, FZD-1, HNF4alpha, LXR ligand RA, Nrh1, C-ERBA in WT hearts; and APOC3, ERK1/2, MEFV, NLRP2, p38 MAPK, S1PR, S1PR4, S1P, GDF15, NF-kB in mIGF-1 Tg hearts), of immunity (C3, XBP1 in WT hearts; DHX58, ERK1/2, G protein alpha, GPCR, GPR27, HLA-A, IFNA1/IFNA13, p38 MAPK, PYCARD, S1P, HLA-abc, IFI30, NF-kB in mIGF-1 Tg hearts), of behavior (IMPA1, KLF-9, PLC, TRPA1 in WT hearts; and ADR2A, ERK1/2, G protein alpha, GPCR, GPR27, NPBWR1, p38 MAPK, NF-kB in mIGF-1 Tg hearts) and of other basic and pathological functions (ABCB11, CALB1, FANCA, FANCL, FZD-1, MYB-2, MYH9, PEX-1, SDF2L1, SLC26A5, SPAM1, SYNE, THRA in WT hearts; and CHRM3, CHRM4, CTAG1B, HIST4H4, PARP10, XIST, Histone H3, KDM3A in mIGF-1 Tg hearts).

**Figure 3:**
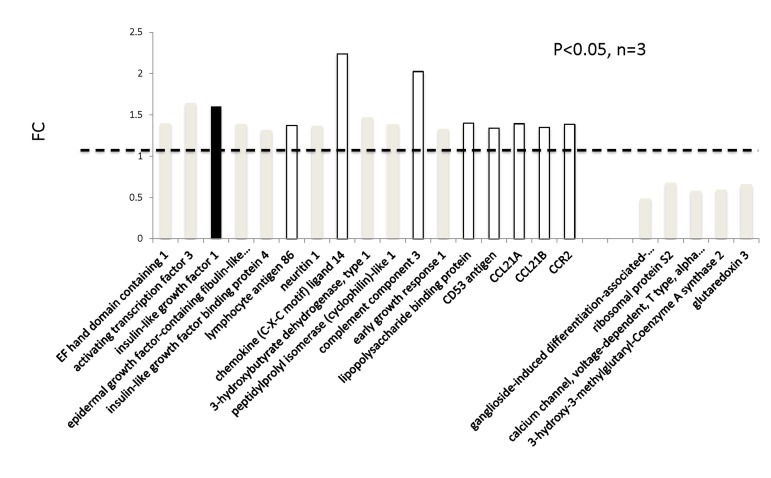
Transcriptomic analyses on the hearts of mIGF-1 Tg versus WT mice The hearts of 3 WT and 3 cardiac-restricted mIGF-1 Tg mice were excised and RNA was processed for Affymetrix Gene ChIP analyses. Data were analyzed using GeneSpring software. Represented genes are those showing fold changes (FC) starting from 1.3 and a p-value lower than 0.05.

**Figure 4:**
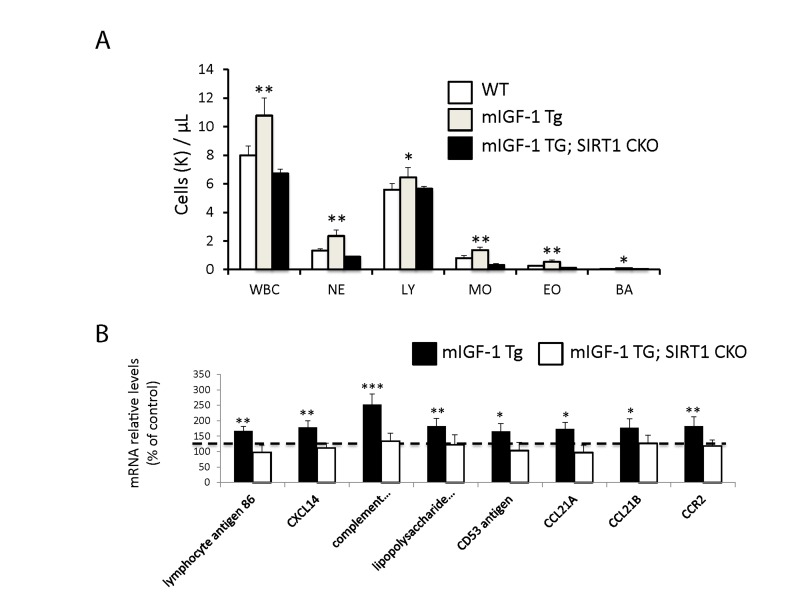
Leukocytosis and cardiac expression of genes involved in the immune response (**A**) Blood was extracted from the tail vain of WT, mIGF-1 Tg mice and mIGF-1; SIRT1 CKO mice and was processed for Hemavet analysis to quantify WBC types: neutrophiles (NE), lymphocytes (LY), monocytes (MO), eosinophiles (EO) and basophiles (BA). Results are means ± SE of 5-11 animals for each genotype (*,** p versus WT mice). (**B**) The expression levels of lymphocyte antigene 86, CXCL14, C3, CD53 antigen, CCL21A, CCL21B and CCR2 mRNAs were examined by Real Time-PCR in the heart of WT, mIGF-1 Tg and mIGF-1 Tg; SIRT1 CKO mice. Results are means ± SE of 5 animals for each genotype (*,**p versus WT mice).

In addition to genome-wide cardiac binding pattern of SIRT1, we sought to determine differences in the transcriptional profiling induced by mIGF-1 transgene in the mouse heart. Previous transcriptomic analyses in the heart of mIGF-1 Tg versus WT mice 24 hours after oxidative injury induced specifically by cardiotoxin injection revealed increased levels of the mitochondrial protein UCP1 (uncoupling protein 1), adiponectin and the antioxidant metallothionein 2 [9]. These three proteins are involved in cardiac protection from oxidative stress, and in isolated cardiomyocytes the upregulation of their levels by mIGF-1 transgene was dependent on SIRT1 activity [5, 9]. Basal transcriptomic analysis on the heart of mIGF-1 Tg versus WT mice in absence of any injury has not been performed and could help to explain why the cardiomyocytes of these mice respond better to specific types of stress (ischemia, angiotensin II, cardiotoxin injection, paraquat injection), with consequent re-generation of the heart tissue [6, 9, 10]. To this purpose, RNA from WT and mIGF-1 Tg hearts was processed for Affymetrix GeneChip analysis. Interestingly, we found that only 22 genes showed a >30% change in mRNA levels (17 upregulated and 5 downregulated, respectively, p<0.05) in the hearts of mIGF-1 Tg mice compared to WT littermates (Fig. 3). Transcript for IGF-1 was found upregulated as expected (Fig. 3, black bar). Most of the upregulated genes in mIGF-1 Tg hearts, 8 out of 17, pertain to the regulation of immune function: lymphocyte antigene 86 (a protein playing an important role in Toll-like receptor 4 pathway), chemokine (C-X-C motif) ligand 14 (a chemoattractant targeting tissue macrophages), C3 (complement component 3, protein whose activation is required for both classical and alternative complement activation pathways, whose promoter is bound by SIRT1, see above), lipopolysaccaride binding protein, CD53 antigen (a tetraspanin protein of the lymphoid-myeloid lineage), CCL21A/CCL21B (potent chemoattractants for lymphocytes) and CCR2 (receptor for monocyte chemoattractant protein-1, MCP-1) (Fig. 3, open bars). The upregulation of these 8 genes was confirmed by qRT-PCR (Fig. 4B) and indicates that cardiac restricted mIGF-1 transgene, in absence of stress, could activate yet uncharacterized immune cell types and mechanisms.

We report thus that at the basal state mIGF-1/SIRT1 genomic and transcriptomic effects in mouse cardiomyocytes are not only related to cardioprotection and anti-oxidant defenses observed under stress conditions [5, 6, 9]. Instead, comparison of both ChIP-Seq data for SIRT1 genome binding and transcriptomic data between WT and mIGF-1 Tg hearts revealed unexpected targets that point to regulatory functions of the mIGF-1/SIRT1 signaling beyond cardiomyocyte specific mechanisms. This led us to perform a more in depth phenotyping and behavioral analysis of these mice, looking closer at three major body compartments involved in stress and aging: the immune system, the arterial blood pressure control system and the behavioral responses dependent on the central nervous system.

### Blood leukocytosis and increased arterial blood pressure levels in cardiac restricted mIGF-1 Tg mice

**Figure 5:**
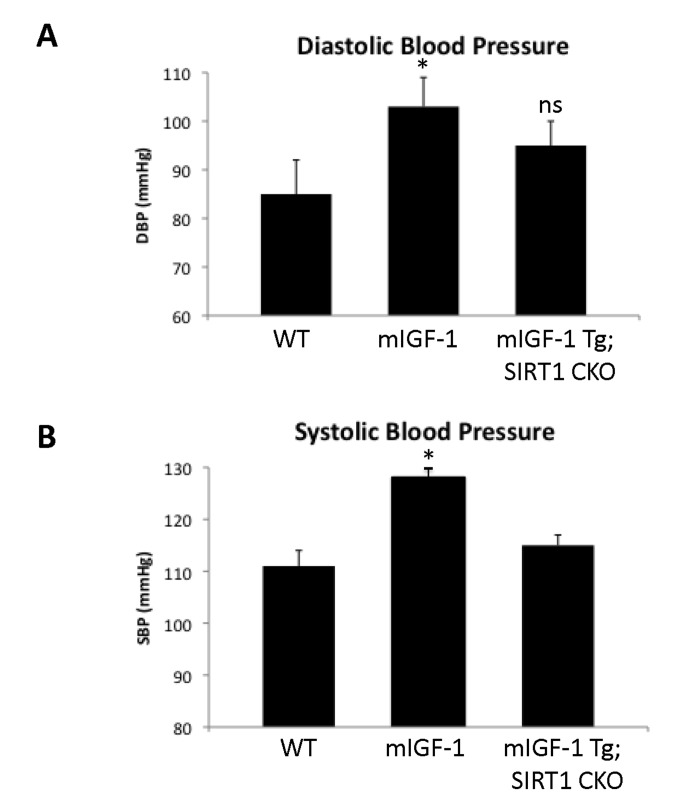
Blood pressure in WT, mIGF-1 Tg and mIGF-1 Tg; SIRT1 CKO mice Noninvasive blood pressure values in conscious mice were measured using the tail-cuff method. (**A**) Diastolic blood pressure (DBP); (**B**) Systolic blood pressure (SBP). Results are means ± SE of 3-8 animals for each genotype (*p versus WT mice). NS= not significant.

Since ChIP-Seq analysis of SIRT1 genome-wide binding in the mouse heart identified several promoters of genes involved in the immune function, and transcriptomic analysis pointed to an upregulation of 8 key genes implicated in the immune response, we sought to quantify accurately the white blood cell (WBC, leukocytes) number in the peripheral blood of WT and mIGF-1 Tg mice. Blood was collected from WT and mIGF-1 Tg mice through the tail vein and the five different types of leukocytes were measured by hematological profile using the Hemavet blood analyzer. We found that the total count of WBC was significantly increased in the blood of mIGF-1 Tg mice compared to WT mice (Fig. 4A). This total increase in WBC was reflected in a significant increase of all the individual types of leucocytes analyzed (neutrophiles, lymphocytes, monocytes, eosinophiles and basophiles), in terms of thousands of cells per μl (Fig. 4). If values were represented as percentage of cells compared to the total of WBC, mIGF-1 Tg mice displayed 10% less lymphocytes than WT mice, although this difference was not statistically significant (data not shown). To understand if mIGF-1 cardiac transgene induced leukocytosis was dependent on SIRT1 activity, we analyzed the blood of mIGF-1 Tg where SIRT1 activity was specifically ablated in a conditional (αMHC-Cre) and tamoxifen-inducible manner in cardiomyocytes (mIGF-1 Tg; SIRT1 CKO), as we have recently described [6]. This maneuver restored WBC to WT level, indicating that SIRT1 is indispensable for the increase in WBC count observed in cardiac restricted mIGF-1 Tg mice (Fig. 4A). The WBC count of SIRT1 CKO was indistinguishable from WT mice (data not shown). Further, we analyzed by qRT-PCR the cardiac expression of the 8 genes that we found upregulated in Affymetrix analysis in the heart of mIGF-1 Tg mice (lymphocyte antigene 86, CXCL14, C3, CD53 antigen, CCL21A, CCL21B, CCR2) (Fig. 3). A significant upregulation of these genes was confirmed in the heart of mIGF-1 Tg mice compared to WT mice, and it occurred to a similar extent when compared to the one detected by microarrays (Fig. 4B). SIRT1 CKO hearts had similar levels of these transcripts as of WT (data not shown). However, genetic deletion of SIRT1 abrogated the immune gene expression signature observed in mIGF-1 Tg mice (Fig. 4B). These results suggest that SIRT1 is the major transducer whereby mIGF-1 stimulates the expression of immune-related genes in the heart tissue. A raise in total WBC count above the normal range is frequently a sign of an inflammatory response or of a generic stress [25]; moreover, in the elderly leukocytosis has been observed in association to an increased incidence of ischemic cardiovascular events [26]. As stated, mIGF-1 Tg mice are protected from oxidative and hypertrophic stresses and do not display any evident morphological feature of cardiac dysfunction as detected by histology or echocardiography [6, 9]. The latter results, together with the finding that SIRT1 binds promoter of genes involved in cardiovascular function and blood pressure control (ABCG1, C3, CTGF, MYH11, PDGF, TRPA1 in WT hearts; and ADRB1, ERK1/2, G protein alpha, GPCR, p38 MAPK, VEGFB, NF-kB in mIGF-1 Tg hearts) (Fig. 1), prompted us to measure in these animals blood pressure, a direct output of cardiac performance. We non-invasively measured the diastolic and systolic blood pressure (DBP and SBP, respectively) in a cohort of WT, SIRT1 CKO, mIGF-1 Tg and mIGF-1 Tg; SIRT1 CKO using the well-established tail-cuff method. While no difference was observed between WT and SIRT1 CKO mice, these analyses showed a ~20-25% significant increase in both DBP and SBP in mIGF-1 Tg mice compared to WT littermates, in the basal state (Fig. 5). This reproducible increase in diastolic blood pressure (DBP) and systolic blood pressure (SBP) induced by cardiac restricted mIGF-1 transgene could be defined as mild hypertension, as in genetic and dietary murine models of hypertension the values of DBP and/or SBP can reach a 2-fold difference [27]. When blood pressure was analyzed in mIGF-1 Tg; SIRT1 CKO mice, DBP showed a trend of increase compared to WT, although this was not significant and was to a lesser extent when compared to mIGF-1 Tg mice (Fig. 5); SBP on the other hand was found restored to WT level in mIGF-1 Tg; SIRT CKO mice (Fig. 5). These data suggest a mild hypertension in cardiac overexpressing mIGF-1 Tg mice, which could at least in part be rescued by cardiomyocyte-specific inactivation of SIRT1 enzyme.

### Increased response to fear in mIGF-1 Tg mice

**Figure 6:**
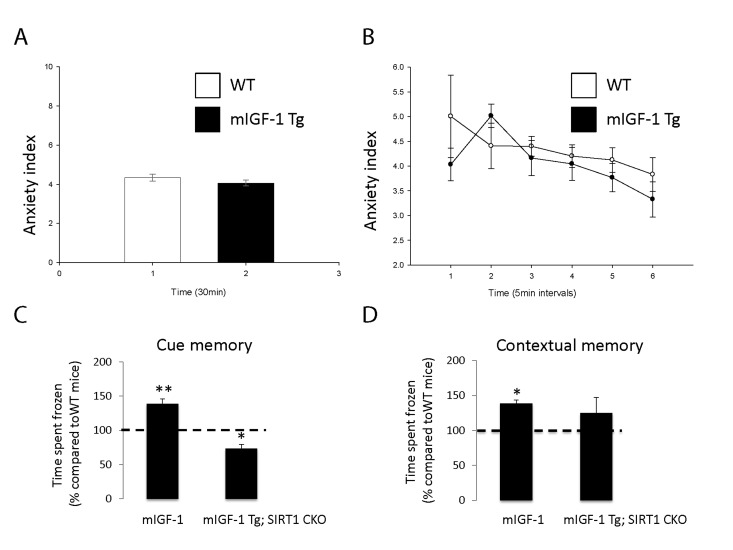
Open field test and fear conditioning test in WT, mIGF-1 Tg and mIGF-1 Tg; SIRT1 CKO mice (**A**, **B**) WT or mIGF-1 Tg mice were subjected to open field test. (**C**, **D**) WT, mIGF-1 Tg and mIGF-1 Tg; SIRT1 CKO mice underwent cue memory or conditional memory conditioning tests. Results are means ± SE of 3-8 animals for each genotype (*,**p versus WT mice).

It is well established that the effect of stress on the immune system and arterial blood pressure is reciprocally linked to individual and social behavioral responses (such as fear, anxiety) in animal models and human beings [28-30]. To determine if cardiomyocyte specific mIGF-1 transgene could impact on behavioral stress responses, and to understand the role of downstream SIRT1 in this process, we ran in our mice open field and fear conditioning (FC) tests, to determine anxiety levels and responsiveness to an aversive stimulus, respectively. The open field test is commonly used in mice as a qualitative and quantitative measure of locomotor activity and willingness to explore. When WT were compared to mIGF-1 Tg mice, there was no statistical difference in the emotionality/anxiety index if the animals were observed either at the end of the test (30 min, Fig. 6A) or at 5 minute intervals (Fig. 6B). Therefore mild elevated arterial blood pressure in mIGF-1 Tg mice is not overtly linked to anxious behavior. Next, we performed FC test. FC provides a measure of memory of the association between an aversive stimulus such as a mild foot shock and an environmental input. The environmental input consists in a discrete stimulus, i.e. a tone or the test chamber (context). In the FC test, freezing behavior, which is a characteristic fear response in mice (total lack of movement), provides a read-out of memory. Animals that show good memory freeze upon re-presentation of the context (contextual fear conditioning) or the tone (cued fear conditioning). Aging has been reported to decrease memory to an aversive stimulus (decreased freezing) in context- and cue-dependent fear response [31-34]. FC test on WT and mIGF-1 Tg mice showed that mice harboring mIGF-1 transgene in the heart had a better memory of the aversive stimulus, for both cue and context fear response, in that they spent a significant augmented time in a frozen position compared to WT littermates with both environmental inputs (Fig. 6C and D). We then performed FC test in mIGF-1 Tg mice devoid of SIRT1 in cardiomyocytes (mIGF-1 Tg; SIRT1 CKO): these mice surprisingly had a decreased cue-dependent fear response compared to WT, whereas the context-dependent fear response was not significantly altered (Fig. 6C and D). In conclusion, the overexpression of mIGF-1 transgene in mouse heart impinges on the behavior of the whole animals and ameliorates their memory of an aversive stimulus, assessed as freezing in response to fear, and this occurs in a SIRT1 dependent manner.

## DISCUSSION

Recently, using Angiotensin II and paraquat as oxidative stressors, we identified an important signaling pathway that protects cardiomyocytes and relies on the activation of SIRT1 by the locally acting mIGF-1 isoform [5, 6]. Cardiac-specific mIGF-1 Tg mice in which SIRT1 was depleted from adult cardiomyocytes confirmed that this pathway is necessary to protect the heart from paraquat-induced oxidative stress and lethality [6]. SIRT1 deacetylates both cytoplasmic and nuclear proteins, in turn regulating several transcrip-tional factors [4]: to understand the mechanisms of mIGF-1/SIRT1 dependent cardio-protection, it was important to assess where SIRT1 is located within the cardiomyocytes. In contrast with recent reports [21, 22], where high resolution microscopy and specific markers were not used, we have found here that SIRT1 is located in the nucleus of a fraction of cardiomyocytes, whereas it is located in the cytoplasm of non-cardiomyocyte cell types. In order to understand how SIRT1 might regulate gene expression, ChIP-Seq offered us a powerful tool to determine how SIRT1 interacts with cardiomyocyte DNA. To our knowledge this is the first SIRT1 genome-wide DNA binding data set reported, after the one obtained by Oberdoerferr et al. in embryonic stem (ES) cells with a ChIP-on-ChIP approach [20]. In accordance with this previous report, the majority of reads for SIRT1 bound DNA accounted for repetitive regions of the genome; this pattern was not any different in WT or in mIGF-1 Tg hearts (data not shown). Oberdoerferr et al. reported a few hundreds of genes bound by SIRT1 in the promoter region in ES cells under basal conditions, and in conditions of oxidative stress (H2O2 treatment) there was a massive displacement of SIRT1 that goes to occupy other promoters [20]. In our system, SIRT1 was found similarly bound to 302 gene promoters, but there were only few dozens of promoters exclusively bound by SIRT1 either in the WT heart or in the mIGF-1 Tg heart (Fig. 2). This distribution indicates that the mIGF-1 transgene induces only subtle alterations in SIRT1 DNA binding patterns. Subtle variations were found also in the microarrays, with only 22 genes showing a significant change in mRNA levels in the mIGF-1 Tg background. Most interestingly these variations in the mIGF-1/SIRT1 genomic and transcriptomic effects in unchallenged mouse hearts were related to genes regulating functions beyond cardiomyocyte specific homeostatic and protective mechanisms. For instance we found genes implicated in the immune response, the blood pressure control, inflammation and behavior. We thus suspected that the cardioprotective mIGF-1/SIRT1 pathway might elicit a global impact on body functions.

Our finding that mIGF-1 mice present in a SIRT1 dependent manner mild increased count of WBC, deriving from multipotent hematopoietic stem cells in the bone marrow, is consistent with the mIGF-1 dependent mobilization of cell populations in the bone marrow that can be primed to repair the injured heart [10]. In absence of any challenge, a “primed” immune system can constitute an advantage for responding to cardiac tissue damage. SIRT1 specifically inhibits Foxp3+ T-regulatory cells [35], which have a role in cardiac remodeling after oxidative damage [36]; dissecting the role of mIGF-1/SIRT1 in boosting specific B-cell and T-cell population under basal conditions or challenge will be of the foremost interest. The fact that mIGF-1 Tg mice were mildly hypertensive was somewhat surprising, considering that blood pressure is only partially determined by the heart, but mostly by peripheral vascular resistance. This effect could be rescued by cardiomyocyte specific depletion of SIRT1 and was not associated to a decreased cardiovascular performance [6]. Plasma measurement of vasoactive hormones associated to immune stress, elevated blood pressure and cognitive functions, such as corticosterone and vasopressin, did not show any change (data not shown), indicating a lack of stress. Accordingly, mIGF-1 Tg mice did not display anxious behavior in the open field test. Instead, they perform much better in a FC test, i.e. they remember better than WT an aversive stimulus and spend more time freezing. This was strictly dependent on the presence of SIRT1: its ablation from cardiomyocytes even worsened the cue fear response (Fig. 6). Contextual and cue fear conditioning are dependent upon the integrity of the hippocampus and the basolateral amygdala, regions of the brain ancestrally connected to changes in heart rate and blood pressure. Although the consensus is that hypertension favors a decline in memory, a study in centenarians showed conversely that high blood pressure is associated to improved cognitive functions [37]. The liaison between high blood pressure triggered by cardiac mIGF-1 transgene, downstream SIRT1 and improved memory of an aversive stimulus remains unclear.

**Figure 7:**
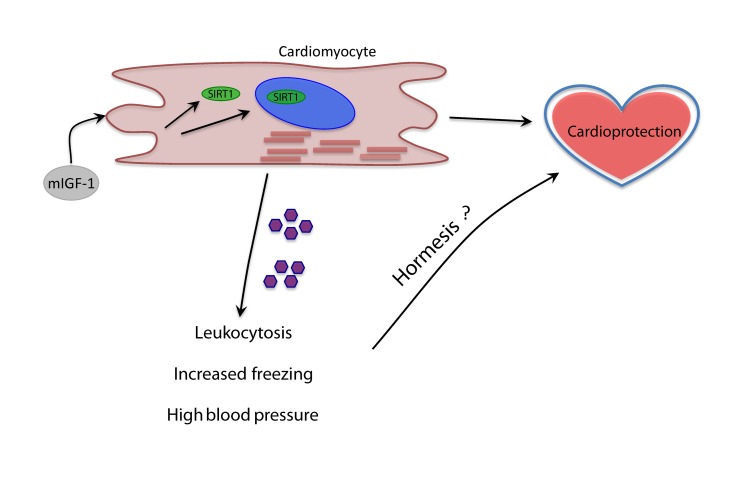
Model of the systemic effects of cardioprotective mIGF-1/SIRT1 pathway Purple pentagons represent circulating factors yet to be identified (question mark).

Of note, we found increased mRNA levels of chemokines CCL21A and CCL21B in the heart of mIGF-1 Tg mice. A study from Villeda et al. recently showed that increasing peripheral CCL11 chemokine levels in vivo in young mice impaired memory in the FC test [38]. This was originally implicating a member of the CCL family in the regulation of cognitive functions, and it will be of interest to assess if high circulating levels of CCL21A/CCL21B might play a role in the observed improved cognitive function of mIGF-1 Tg mice. Recent breakthrough data show for the first time that modulation of a single cardiac signaling pathway have profound effects on global energy homeostasis: inhibition of miR-208 in the heart of mice confers resistance to high-fat diet-induced obesity, and improves systemic insulin sensitivity and glucose tolerance [39]. Here we propose that it can also influence globally the immune system, arterial blood pressure and even an ancient behavioral mechanism such as the response to fear. The identity of the circulating factors, such as cytokines and metabolites, and mechanisms mediating the systemic response of cardiac specific mIGF-1/SIRT1 remain to be addressed (Fig. 7). From the point of view of biogerontology, the impact of cardioprotective mIGF-1 pathway on immune and cardiovascular stress is coherent with the concept of hormesis, i.e. exposing cells and organisms to mild stress should result in an adaptive response with various benefits (Fig. 7) [40-42]. We did not perform yet a longitudinal study of life span in mIGF-1 Tg mice to determine if they live longer, although they are functionally as healthy as the wild type and protected against potent oxidative stressors hitting the heart or the whole body [5, 6, 9, 10]. Moreover, the improved memory in remembering an aversive stimulus of mIGF-1 Tg mice is opposite to what is observed in old animals [31-34, 38]. Elucidating in detail the systemic effects of the cardioprotective mIGF-1/SIRT1 pathway may help to develop new cardiac and anti-aging therapies.

## METHODS

### Animal models

Transgenic FVB mice carrying a rat mIGF-1 cDNA driven by the mouse αMyHC promoter (αMyHC/mIGF-1) were generated and maintained as previously described [9]. SIRT1 floxed (Fl/Fl) mice were previously described [14] and acquired from The Jackson Laboratory. Tamoxifen-inducible αMyHC/mER-CRE-mER transgenic mice were crossed to SIRT1 floxed (Fl/Fl) mice to deplete SIRT1 expression in adult CM [43] upon tamoxifen administration. Mice were placed on tamoxifen-containing chow (Harlan Special Diet TD.55125) at 4 months of age, for two weeks, leading to efficient and reproducible gene recombination [44]. The mIGF-1 transgene was introduced by three-way crosses to generate αMyHC/mIGF-1 Tg × αMyHC/mER-CRE-mER; SirT1Fl/Fl mice (referred to as mIGF-1 Tg × SirT1 CKO) [6]. PCR genotyping was performed using genomic DNA from tail biopsies.

### Immunofluorescence

WT and mIGF-1 Tg mice were sacrificed by cervical dislocation, and hearts were perfused by injecting PBS through the right ventricle. Mice hearts were cryopreserved in OCT embedding matrix (Cellpath), snap freeze in liquid nitrogen (N2), and stored at −80°C. Semi-thinsections (7μm) were cut at −20°C with the help of a cryostat (Leica Microsystems, Mannheim) and fixed with 4% Paraformaldehyde (PFA) (w/v) in PBS for 20 min. at RT, and then processed for blocking and immunolabelling. Sections were quenched for 10 min. with 100mm NH4Cl, permeabilized for 10 min. with 0.5% (v/v) Triton X100 and blocked for 1h with (10% (w/v) BSA, 0.1% fish gelatin (Sigma) in PBS. Subsequently sections were incubated with primary antibodies diluted in 0.5% BSA, 0.1% gelatin, PBS (PBG) overnight at 4°C and with secondary antibodies diluted in PBG for 2 h at room temperature. Nuclei were visualized with DAPI coloration, and sections were mounted with Prolong Gold medium (Life Technologies).

### Confocal microscopy

Sections were observed with a laser scanning confocal microscope, TCS SP5 (Leica Microsystems, Mannheim) using a 40x (NA=1.25) lens with optical pinhole at 1AU. A UV laser operating at 405 nm, an Argon 488 nm wavelength and a HeNe 543 nm were used as excitation sources. Optical sections of 0.4 μm were acquired and processed with Volocity software (Improvision, Perkin Elmer) for image rendering and measurements. For quantitative analyses of SIRT1 positive cardiomyocytes, cell nuclei in 10 random fields of 10 different sections were processed through a Volocity based protocol.

### Chromatin immunoprecipitation sequencing (ChIP-Seq)

Protein-DNA complexes were captured by fixing heart homogenates from adult wild type or mIGF-1 Tg mouse at resting state in 1% (v/v) formaldehyde (Sigma) for 10 min at room temperature with gentle shaking. The reaction was quenched by adding 0.125M glycine for 5 min at room temperature. Cells were washed (x3) with cold PBS and resuspended in Cell Lysis Buffer (CLB) (50mM Hepes, 140mM NaCl, 1mM EDTA, 10% glycerol, 0.5% NP40, 1x proteinase inhibitor; 1 x107 cells/ml) on ice for 10min to release nuclei. Nuclei were pelleted (4000 rpm, 10 min, 4 C) and resuspended in Nuclei Lysis Buffer (NLB) (140mM NaCl, 10mM Tris-Cl (pH 8), 1% NP40, 1x proteinase inhibitor) on ice for 10 min. Cells were sonicated using the Bioruptor Sonicating Waterbath (Diagenode). 300 ml of lysate was sonicated for 40 pulses of 30s ON 30s OFF in 1.5 ml TPX eppendorf tubes, replacing ice regularly to minimize overheating of samples. Chromatin was pooled and centrifuged for at 13000 rpm, 10 min, 4 C to remove debris and single use aliquots (300ul) were used for immunoprecipitation or stored at -80 C. Chromatin was immunoprecipitated by bringing each aliquot of chromatin to 1ml with NLB proteinase inhibitor. Chromatin was precleared with 80ul of ChIP-grade Protein Agarose A beads (Upstate) for 4 hr at 4 C with rotation and incubated overnight with 4 mg of SIRT1 monoclonal antibody (Mount Sinai Antibody Facility, NY), or unrelated rabbit IgG polyclonal antibody (Chemicon) at 4 C with rotation. Immunoprecipitated complexes were collected by incubation with 100 μl of ChIP blocked Protein A agarose beads (Upstate) for 4hr at 4 C with rotation and non-specific complexes were removed by washing beads twice with High Salt Buffer (0.1% SDS, 1% Triton-X, 2mM EDTA, 20mM Tris-Cl (pH 8.1), 500mM NaCl), Low Salt Buffer (0.1% SDS, 1% Triton-X, 2mM EDTA, 20mM Tris-Cl (pH 8.1), 150mM NaCl), LiCl Buffer (0.25M LiCl, 1% NP40, 1% deoxycholic acid, 1mM EDTA, 10mM Tric-Cl (pH 8.1) and TE Buffer. Washes were performed for 5min at 4 C with rotation and beads were collected by centrifugation at 3000 rpm, 4 C 3 min. DNA was eluted with 100 μl of Elution Buffer (1% SDS, 100mM NaHCO3). Beads were vortexed for 30 s and incubated at room temperature with rotation for 15min and the process was repeated and eluates combined (200 μl). Crosslinks were reversed with 0.3M NaCl by incubating at 65 C overnight in a hybridization oven and RNA was removed with RNase A (Upstate) at 37 C for 30 min. Protein was removed by incubating samples with Proteinase K (Upstate) for 1 hr at 45 C. DNA was purified using Qiagen PCR Purification Kit (QIAGEN) and eluted in 30 μl of dH20. For ChIP-seq individual SIRT1 ChIP samples were pooled and concentrated using a SpeedVac. The ChIP templates were sequenced using a Solexa Gene Analyzer II platform (Illumina) at 36 bp read length using standard manufacturer protocols at the Genomics Core Facility in Heidelberg, Germany.

### ChIP-Seq data analysis

Data alignment: ChIP-sequencing data, fastq files, (Mock, 1 sample; IGF overexpression, 2 samples; WT, 2 samples) were aligned against the mouse unmasked genome (NCBI mm9) using the SHRIMP alignment software [45]. We obtained the following alignments: Mock 78.9% mapped reads (7.9 millions); IGF overexpression: IGF1a 94.4% (28.2 millions), IGF1b 82.7% (21.0 millions); WT: WT1 87.4% (10.0 millions), WT2 95.4% (27.1 millions).

Data segmentation and filtering: Data segmentation was done, chromosome by chromosome using BayesPeak algorithm [46], implemented in the Bioconductor package BayesPeak. As background the Mock sample was used. Peaks selection was done using thresholds based on the posterior probability generated by the software, PP≥0.5, subsequently only the peaks overlapping in replicated experiment were considered. This approach yielded 5347 WT peaks and 9467 IGF1 peaks that were merged.

Peaks were annotated using ChIPpeakAnno package [47] and only genes characterized by a potential SIRT1 binding within 10Kb from the gene start site were considered. Furthermore 5305 peaks were left removing all peaks with less than 10 reads/peak. Subsequently 302 peaks were kept upon removal of all peaks that were characterized by having less than 3 times the number of counts of the corresponding genomic region in the Mock sample.

### Affymetrix analysis

RNA from WT and Tg hearts was processed for Affymetrix GeneChip analysis by the GeneCore Facility at EMBL (EMBL, http://www.genecore.embl.de/), and a mouse 430A 2.0 chip was used for the analysis. Data were processed by GeneSpring.

### Blood pressure measurement

Non-invasive BP was evaluated by tail-cuff measurements (Visitech Systems, Apex, NC, USA) as described previously [48].

### Behavioral tests

*Fear conditioning test*: Adult mice (2- 4 months of age) were trained and tested in an operant chamber (18.5 × 18 × 21.5 cm) possessing aluminum sidewalls and Plexiglas rear and front walls (Coulbourn Instruments). The auditory cue (tone) emanated from a loudspeaker located in the sidewall, 15 cm from the floor. The activity of animals was recorded directly on a computer through a single camera located at the top of the chamber. The presentation of tone and shock stimuli in all training and testing sessions was controlled by GraphicState software (Coulbourn Instruments). The chamber was cleaned with ethanol and dried between mice after each training or testing session. Conditioning: mice were allowed to acclimate to the training chamber for 2 min, and then a tone (CS) of 2800 Hz frequency and 85 dB intensity was presented for 30 s and coterminated in the last 2 s with a mild footshock (0.5 mA) [unconditioned stimulus (US)]. Two minutes later, another CS-US pairing was presented, and the mice were returned to their home cages 30 s later.

To test for a conditioned fear response to context, the mice were placed in the same chamber used for the conditioning trial 24 h after conditioning and allowed to explore for 4 min without presentation of the auditory CS or footshock. Freezing behavior, defined as complete absence of voluntary movements except for respiratory movements, was scored every 1 s. Twenty-four hours after the contextual conditioning test, the mice were tested for cue memory by returning them to the same chamber, which was modified by the addition of various shapes and designs on the walls and a vanilla scent. Freezing behavior was scored for 2 min before delivery of the tone and then for 2 min in the presence of a continuous auditory cue. Freezing behavior was measured with an automated system whereby continuous video data were analyzed with the aid of Freeze-frame software (Coulbourn Instruments). The software measures the variance in pixel intensity across successive video frames (taken at 1 Hz) and computes its SD. A threshold is then applied to the data to yield a percentage freezing score. Two-way ANOVA tests (with genotype as a between-subjects factor and context or tone tests as a within-subject factor) were used for statistical analysis with p < 0.05 set as the criterion for statistical significance. Post hoc group comparisons were performed with Fisher's LSD test.

*Open Field:* mice were placed at the periphery of the open field apparatus (consisting of a homogeneously and indirectly illuminated angular arena with walls made of plastic material) with the head facing toward the proximal wall and allowed to explore the arena freely for 30 min. The experimenter was out of view from the mice at all times. The distance traveled and the time spent in the central and peripheral regions were automatically recorded on either a video-tracking system or infrared sensors. The percentage of time spent in the central zone was used as index of emotionality/anxiety.

### Immune Cell Count

Count of white blood cells (WBC) was performed using a Hemavet 950 device, according to manufacturer instructions (Drew Scientific, United Kingdom).

### Real-Time PCR

Total RNA was isolated from hearts using TRIzol (Invitrogen). After RNA quality verification, 1-2 mg was used to prepare cDNA (Ready-To-Go, T-Primed First-Strand Kit, Amersham Bioscience). Quantitative polymerase chain reaction (PCR) for SirT1 was performed using the SYBR Green (SIGMA) in a Light-Cycler (Roche). Primer sequences were: lymphocyte antigene 86 Forward: AGTGGGGGCTTGGAAGTAGT, Reverse: AGAAGAGCCTTTTGCCATCA; CXCL14 Forward: CTCCAGGCCAGTTGAGAGAC, Reverse: CTGGAAGCCTTTCACACACA; C3 Forward: AAGCATCAACACACCCAACA, Reverse: CTTGAGCTCCATTCGTGACA; CD53 antigen Forward: CAGTGGTCCACCATCTTCCT, Reverse: GCAAGTCACAGCCCTAAAGC; CCL21A Forward: ATGTGCAAACCCTGAGGAAG, Reverse: TCCTCTTGAGGGCTGTGTCT; CCL21B Forward: GGGCTGCAAGAGAACTGAAC, Reverse: CCGTGCAGATGTAATGGTTG; CCR2 Forward: AGAGAGCTGCAGCAAAAAGG, Reverse: GGAAAGAGGCAGTTGCAAAG. UbiC, Rn18S and GAPDH transcripts were used as internal controls, according to the GeNorm method [5].

### Statistical analysis

Results are expressed as means ± S.E. Comparisons were made by using Student's t test. Differences were considered as significant when P<0.05 (*), P<0.01 (**) or P<0.001 (***).

### Reagents and antibodies

All reagents not described elsewhere in the text are listed in Supplemental Table I.

## SUPPLEMENTAL MATERIALS



## References

[R1] Fontana L, Vinciguerra M, Longo VD (2012). Growth factors, nutrient signaling, and cardiovascular aging. Circ Res.

[R2] Guarente L, Franklin H (2011). Epstein Lecture: Sirtuins, aging, and medicine. N Engl J Med.

[R3] Kenyon CJ (2010). The genetics of ageing. Nature.

[R4] Vinciguerra M, Fulco M, Ladurner A, Sartorelli V, Rosenthal N (2010). SirT1 in muscle physiology and disease: lessons from mouse models. Dis Model Mech.

[R5] Vinciguerra M, Santini MP, Claycomb WC, Ladurner AG, Rosenthal N (2009). Local IGF-1 isoform protects cardiomyocytes from hypertrophic and oxidative stresses via SirT1 activity. Aging (Albany NY).

[R6] Vinciguerra M, Santini MP, Martinez C, Pazienza V, Claycomb WC, Giuliani A, Rosenthal N (2012). mIGF-1/JNK1/SirT1 signaling confers protection against oxidative stress in the heart. Aging Cell.

[R7] Vinciguerra M, Musaro A, Rosenthal N (2010). Regulation of muscle atrophy in aging and disease. Adv Exp Med Biol.

[R8] Winn N, Paul A, Musaro A, Rosenthal N (2002). Insulin-like growth factor isoforms in skeletal muscle aging, regeneration, and disease. Cold Spring Harb Symp Quant Biol.

[R9] Santini MP, Tsao L, Monassier L, Theodoropoulos C, Carter J, Lara-Pezzi E, Slonimsky E, Salimova E, Delafontaine P, Song YH, Bergmann M, Freund C, Suzuki K, Rosenthal N (2007). Enhancing repair of the mammalian heart. Circ Res.

[R10] Santini MP, Lexow J, Borsellino G, Slonimski E, Zarrinpashneh E, Poggioli T, Rosenthal N (2011). IGF-1Ea induces vessel formation after injury and mediates bone marrow and heart cross-talk through the expression of specific cytokines. Biochem Biophys Res Commun.

[R11] Lagouge M, Argmann C, Gerhart-Hines Z, Meziane H, Lerin C, Daussin F, Messadeq N, Milne J, Lambert P, Elliott P, Geny B, Laakso M, Puigserver P, Auwerx J (2006). Resveratrol improves mitochondrial function and protects against metabolic disease by activating SIRT1 and PGC-1alpha. Cell.

[R12] Howitz K, Bitterman KJ, Cohen HY, Lamming DW, Lavu S, Wood JG, Zipkin RE, Chung P, Kisielewski A, Zhang LL, Scherer B, Sinclair DA (2003). Small molecule activators of sirtuins extend Saccharomyces cerevisiae lifespan. Nature.

[R13] Cohen HY, Miller C, Bitterman KJ, Wall NR, Hekking B, Kessler B, Howitz KT, Gorospe M, de Cabo R, Sinclair DA (2004). Calorie restriction promotes mammalian cell survival by inducing the SIRT1 deacetylase. Science.

[R14] Cheng H, Mostoslavsky R, Saito S, Manis J, Gu Y, Patel P, Bronson R, Appella E, Alt F, Chua K (2003). Developmental defects and p53 hyperacetylation in Sir2 homolog (SIRT1)-deficient mice. Proc Natl Acad Sci U S A.

[R15] McBurney M, Yang X, Jardine K, Hixon M, Boekelheide K, Webb JR, Lansdorp PM, Lemieux M (2003). The mammalian SIR2alpha protein has a role in embryogenesis and gametogenesis. Mol Cell Biol.

[R16] Alcendor RR, Gao S, Zhai P, Zablocki D, Holle E, Yu X, Tian B, Wagner T, Vatner SF, Sadoshima (2007). Sirt1 regulates aging and resistance to oxidative stress in the heart. Circ Res.

[R17] Schug TT, Li X (2010). Surprising sirtuin crosstalk in the heart. Aging (Albany NY).

[R18] Stein S, Matter CM (2011). Protective roles of SIRT1 in atherosclerosis. Cell Cycle.

[R19] Tonkin J, Villarroya F, Puri PL, Vinciguerra M (2012). SIRT1 signaling as potential modulator of skeletal muscle diseases. Curr Opin Pharmacol.

[R20] Oberdoerffer P, Michan S, McVay M, Mostoslavsky R, Vann J, Park SK, Hartlerode A, Stegmuller J, Hafner A, Loerch P, Wright SM, Mills KD, Bonni A, Yankner BA, Scully R, Prolla TA, Alt FW, Sinclair DA (2008). SIRT1 redistribution on chromatin promotes genomic stability but alters gene expression during aging. Cell.

[R21] Tanno M, Kuno A, Yano T, Miura T, Hisahara S, Ishikawa S, Shimamoto K, Horio Y (2010). Induction of manganese superoxide dismutase by nuclear translocation and activation of SIRT1 promotes cell survival in chronic heart failure. J Biol Chem.

[R22] Tanno M, Sakamoto J, Miura T, Shimamoto K, Horio Y (2007). Nucleocytoplasmic shuttling of the NAD+-dependent histone deacetylase SIRT1. J Biol Chem.

[R23] Ausoni S, Sartore S (2009). The cardiovascular unit as a dynamic player in disease and regeneration. Trends Mol Med.

[R24] Park P (2009). ChIP-seq: advantages and challenges of a maturing technology. Nat Rev Genet.

[R25] Porth C (2011). White blood cell response.

[R26] Bovill EG, Bild DE, Heiss G, Kuller LH, Lee MH, Rock R, Wahl PW (1996). White blood cell counts in persons aged 65 years or more from the Cardiovascular Health Study. Correlations with baseline clinical and demographic characteristics. Am J Epidemiol.

[R27] Johns C, Gavras I, Handy DE, Salomao A, Gavras H (1996). Models of experimental hypertension in mice. Hypertension.

[R28] Elias JW, Elias MF, Schlager G (1975). Aggressive social interaction in mice genetically selected for blood pressure extremes. Behav Biol.

[R29] Capuron L, Miller AH (2011). Immune system to brain signaling: neuropsychopharmacological implications. Pharmacol Ther.

[R30] Nautiyal KM, Ribeiro AC, Pfaff DW, Silver R (2008). Brain mast cells link the immune system to anxiety-like behavior. Proc Natl Acad Sci U S A.

[R31] Gould TJ, Feiro OR (2005). Age-related deficits in the retention of memories for cued fear conditioning are reversed by galantamine treatment. Behav Brain Res.

[R32] Ohta A, Akiguchi I, Seriu N, Ohnishi K, Yagi H, Higuchi K, Hosokawa M (2001). Deterioration in learning and memory of fear conditioning in response to context in aged SAMP8 mice. Neurobiol Aging.

[R33] Kaczorowski CC, Disterhoft JF (2009). Memory deficits are associated with impaired ability to modulate neuronal excitability in middle-aged mice. Learn Mem.

[R34] Liu R, Liu IY, Bi X, Thompson RF, Doctrow SR, Malfroy B, Baudry M (2003). Reversal of age-related learning deficits and brain oxidative stress in mice with superoxide dismutase/catalase mimetics. Proc Natl Acad Sci U S A.

[R35] Beier UH, Wang L, Bhatti TR, Liu Y, Han R, Ge G, Hancock WW (2011). Sirtuin-1 targeting promotes Foxp3+ T-regulatory cell function and prolongs allograft survival. Mol Cell Biol.

[R36] Tang TT, Yuan J, Zhu ZF, Zhang WC, Xiao H, Xia N, Yan XX, Nie SF, Liu J, Zhou SF, Li JJ, Yao R, Liao MY, Tu X, Liao YH, Cheng X (2012). Regulatory T cells ameliorate cardiac remodeling after myocardial infarction. Basic Res Cardiol.

[R37] Richmond R, Law J, Kay-Lambkin F (2011). Higher blood pressure associated with higher cognition and functionality among centenarians in Australia. Am J Hypertens.

[R38] Villeda SA, Luo J, Mosher KI, Zou B, Britschgi M, Bieri G, Stan TM, Fainberg N, Ding Z, Eggel A, Lucin KM, Czirr E, Park JS, Couillard-Després S, Aigner L, Li G, Peskind ER, Kaye JA, Quinn JF, Galasko DR, Xie XS, Rando TA, Wyss-Coray T (2011). The ageing systemic milieu negatively regulates neurogenesis and cognitive function. Nature.

[R39] Grueter CE, van Rooij E, Johnson BA, Deleon SM, Sutherland LB, Qi X, Gautron L, Elmquist JK, Bassel-Duby R, Olson EN (2012). A Cardiac MicroRNA Governs Systemic Energy Homeostasis by Regulation of MED13. Cell.

[R40] Rattan SI (2008). Hormesis in aging. Ageing Res Rev.

[R41] Gems D, Partridge L (2008). Stress-response hormesis and aging: “that which does not kill us makes us stronger”. Cell Metab.

[R42] Schulz TJ, Westermann D, Isken F, Voigt A, Laube B, Thierbach R, Kuhlow D, Zarse K, Schomburg L, Pfeiffer AF, Tschöpe C, Ristow M (2010). Activation of mitochondrial energy metabolism protects against cardiac failure. Aging (Albany NY).

[R43] Sohal DS, Nghiem M, Crackower MA, Witt SA, Kimball TR, Tymitz KM, Penninger JM, Molkentin JD (2001). Temporally regulated and tissue-specific gene manipulations in the adult and embryonic heart using a tamoxifen-inducible Cre protein. Circ Res.

[R44] Kratsios P, Catela C, Salimova E, Huth M, Berno V, Rosenthal N, Mourkioti F (2010). Distinct roles for cell-autonomous Notch signaling in cardiomyocytes of the embryonic and adult heart. Circ Res.

[R45] Rumble SM, Lacroute P, Dalca AV, Fiume M, Sidow A, Brudno M (2009). SHRiMP: accurate mapping of short color-space reads. PLoS Comput Biol.

[R46] Spyrou C, Stark R, Lynch AG, Tavare S (2009). BayesPeak: Bayesian analysis of ChIP-seq data. BMC Bioinformatics.

[R47] Zhu LJ, Gazin C, Lawson ND, Pages H, Lin SM, Lapointe DS, Green MR (2010). ChIPpeakAnno: a Bioconductor package to annotate ChIP-seq and ChIP-chip data. BMC Bioinformatics.

[R48] Potenza N, Vecchione C, Notte A, De Rienzo A, Rosica A, Bauer L, Affuso A, De Felice M, Russo T, Poulet R, Cifelli G, De Vita G, Lembo G, Di Lauro R (2005). Replacement of K-Ras with H-Ras supports normal embryonic development despite inducing cardiovascular pathology in adult mice. EMBO Rep.

