# Supplementary material for: Cardioprotective mIGF-1/SIRT1 signaling induces hypertension, leukocytosis and fear response in mice
Source: Aging (Albany NY). 2012 Jun 11;4(6):402–16. doi: 10.18632/aging.100464 (PMC3409677; doi:10.18632/aging.100464)
Supplement: Supplementary file 1 [file aging-04-402-s001.pdf]

SUPPLEMENTAL MATERIALS

Supplemental Table 1. Reagents and Antibodies

Primary antibodies:

Protein targeted	Host	Clone	Provider	Catalogue number
SIRT1 (for IF studies)	rabbit		Abcam	Ab12193
MF-20	mouse		DSHB-Iowa	
Vimentin	mouse	V9	Sigma	V6630
CD144	rabbit		Abcam	Ab33168
SIRT1 (for ChIP-Seq)	mouse	10E04	Millipore	NG1693253

Secondary antibodies:

Protein targeted	Host	Provider	Catalogue number
HRP-conjugated anti-mouse	Goat	Amersham – GE Healthcare	NA9310V
HRP-conjugated anti-rabbit	Goat	Amersham – GE Healthcare	NA934V
HRP conjugated anti-Goat	Rabbit	Santa Cruz Biotechnology	sc-2020
Alexa 488 conjugated	Goat	Invitrogen	A-11008
Alexa546 conjugated	Goat	Invitrogen	A-11035

Other reagents:

Name	Provider	Catalogue number
ECL reagent	Amersham – GE Healthcare	RPN2209
Trizol Reagent	Invitrogen	15596
SYBR®Green dye	Sigma	QR0100
Tamoxifen	Sigma	T5648
